# Low Vitamin D Status Attenuates Hypolipidemic and Pleiotropic Effects of Atorvastatin in Women

**DOI:** 10.3390/nu17101674

**Published:** 2025-05-15

**Authors:** Robert Krysiak, Karolina Kowalcze, Witold Szkróbka, Bogusław Okopień

**Affiliations:** 1 Department of Internal Medicine and Clinical Pharmacology, Medical University of Silesia, Medyków 18, 40-752 Katowice, Poland; wszkrobka@sum.edu.pl (W.S.); bokopien@sum.edu.pl (B.O.); 2 Department of Pathophysiology, Faculty of Medicine, Academy of Silesia, Rolna 43, 40-555 Katowice, Poland; kkowalcze@sum.edu.pl; 3 Department of Pediatrics in Bytom, Faculty of Health Sciences in Katowice, Medical University of Silesia, Stefana Batorego 15, 41-902 Bytom, Poland

**Keywords:** calciferol, dyslipidemia, glucose homeostasis, lipids, pleiotropic effects, statins

## Abstract

*Background/Objectives*: Low vitamin D status seems to be associated with increased cardiometabolic risk, and was found to attenuate cardiometabolic benefits of statins in men. The aim of the current study was to investigate whether a different vitamin D status determines the pleiotropic effects of statins in women. *Methods*: This pilot, single-center, prospective, matched-cohort study included 78 women with hypercholesterolemia requiring statin therapy, assigned into one of three age-, plasma lipid-, and body mass index-matched groups: women with vitamin D deficiency (group I), women with vitamin D insufficiency (group II), and women with normal vitamin D homeostasis (group III). Throughout the study (16 weeks), all patients were treated with atorvastatin. The outcome of interest included plasma lipids, glucose homeostasis markers (fasting glucose, HOMA-IR and glycated hemoglobin), plasma levels of 25-hydroxyvitamin D, creatine kinase, uric acid, high-sensitivity C-reactive protein, homocysteine, fibrinogen, urinary albumin-to-creatinine ratio (UACR), and computed values of a 10-year risk of atherosclerotic events. *Results*: Compared to the control group (group III), group I was characterized by higher values of HOMA-IR, glycated hemoglobin, uric acid, hsCRP, homocysteine, fibrinogen, a UACR, and a 10-year risk of atherosclerotic events, whereas group II had higher values of hsCRP, homocysteine and a UACR. Atorvastatin reduced plasma levels of total and LDL cholesterol and a 10-year risk of atherosclerotic events in all study groups, but this effect was weakest in group I and strongest in group III. In group III, the drug decreased uric acid, hsCRP, homocysteine, fibrinogen, and the UACR. In the remaining groups, its effect was limited to a small decrease in only hsCRP (group I) or in hsCRP and homocysteine (group II). In group I, atorvastatin treatment was associated with an increase in HOMA-IR, glycated hemoglobin, and creatine kinase. *Conclusions:* Low vitamin D status may exert an unfavorable effect on the lipid-dependent and lipid-independent effects of atorvastatin in middle-aged or elderly women.

## 1. Introduction

Statins are the most frequently prescribed group of lipid-lowering drugs and one of the most commonly used medications worldwide [1]. They are used in the prevention of cardiovascular events in patients with dyslipidemia, cardiac risk factors, and stable cardiovascular disease [2], the leading cause of morbidity and mortality in developed countries [3]. In addition to reducing low-density lipoprotein (LDL) cholesterol by competitive inhibition of 3-hydroxy-3-methylglutaryl–coenzyme A reductase (HMG-CoA), statins possess many clinically relevant effects that are independent of lipid lowering [4]. These beneficial pleiotropic effects include anti-inflammatory, antioxidant, endothelial-protective, plaque-stabilizing, anti-aggregatory, anticoagulant, profibrinolytic, and immunosuppressive properties, as well as an inhibitory effect on smooth muscle cell proliferation and migration [5,6,7,8,9,10]. Both lipid-lowering and pleiotropic effects seem to contribute to numerous benefits associated with statin therapy, which were documented in large primary and secondary prevention clinical trials [11,12,13,14]. Despite the fact that statins are recommended for the prevention of acute coronary events, stroke and peripheral atherosclerotic events, long-term treatment with these drugs was associated with potentially unfavorable outcomes: increased risk of type 2 diabetes [15] and higher values of the body mass index (BMI) [16].

Both vitamin D receptors and 1α-hydroxylase are present in vascular endothelial and smooth muscle cells, cardiomyocytes, and cardiac fibroblasts [17]. Mice knockout for the vitamin D receptor gene and the 1α-hydroxylase gene were characterized by myocardial hypertrophy, overactivity of the renin–angiotensin–aldosterone system, hypertension, enhanced thrombogenicity, and faster progression of atherosclerosis [18]. Low 25-hydroxyvitamin D concentrations make subjects more susceptible to left ventricle hypertrophy, endothelial dysfunction, and early atherosclerotic changes in the vascular wall (increased thickness of the carotid intima-media complex, increased arterial wall stiffness, and an increased number and size of atherosclerotic plaques in peripheral arteries) [19]. In clinical trials, 25-hydroxyvitamin D levels were inversely associated with all-cause mortality and coronary artery disease mortality [20]. Low vitamin D status was associated with a higher risk of acute myocardial infarction, even after controlling for other cardiovascular risk factors, cardiac remodeling in patients after myocardial infarction, and other post-infarction complications [21]. Moreover, vitamin D was causatively linked to an increased risk of stroke, poor post-stroke outcome, higher risk of death at one or two years following stroke, and to a greater risk of early recurrent stroke [22]. Low serum 25-hydroxyvitamin D levels were also associated with a higher prevalence of peripheral artery disease [23]. Lastly, low circulating 25-hydroxyvitamin D levels were found to predispose to type 2 diabetes, even after adjusting for obesity, physical activity, and other lifestyle parameters, as well as being associated with poorer glycemia control [24]. Each 10 nmol/L increment in 25-hydroxyvitamin D concentration was found to be associated with a 4% lower risk of type 2 diabetes [25]. Calcitriol, the active form of calciferol, decreased renin production, improved relaxation of smooth muscle cells, and inhibited foam cell formation [26]. Moreover, vitamin D supplementation was reported to improve insulin sensitivity, fasting glucose, glycated hemoglobin (HbA_1c_), and plasma lipids [27,28]. Exogenous vitamin D supplementation reduced the incidence of major cardiovascular events, especially myocardial infarction and coronary revascularization, and this protective effect was stronger in individuals treated with statins [29].

Interestingly, calciferol insufficiency was accompanied by impaired cardiometabolic effects of cabergoline and fenofibrate in women [30,31] and by impaired statin effects in men [32]. As far as we know, no previous study has investigated the relationship between statin therapy and vitamin D deficiency or insufficiency in women. Thus, the aim of the current study was to determine whether hypolipidemic and pleiotropic effects of HMG-CoA reductase inhibitors in women requiring statin therapy are influenced by differences in vitamin D status.

## 2. Materials and Methods

Our study was a pilot, single-center, prospective, matched-cohort study. The protocol was approved by the institutional committee on human research. The study was conducted according to the principles of the 1975 Declaration of Helsinki and its subsequent revisions. Informed written consent was obtained from all participants prior to enrollment in the study. Owing to its nature, the study did not have to be registered in the clinical trial registry.

### 2.1. Participants

The participants were recruited among women with elevated total and LDL cholesterol levels (total cholesterol above 190 mg/dL, LDL cholesterol exceeding 115 mg/dL), despite complying for at least 3 months with the lifestyle modification program. To be considered for enrollment, estimated 10-year risk of atherosclerotic cardiovascular disease events had to be between 7.5% and 20%, or between 5% and 7.5% if any risk-enhancing factors were present. Patients’ 10-year risk of cardiovascular death, non-fatal myocardial infarction, and non-fatal stroke was estimated using the online ASCVD Risk Estimator Plus calculator (Available online: https://tools.acc.org/ascvd-risk-estimator-plus/#!/calculate/estimate/, accessed on 12 May 2025), developed and updated by the American College of Cardiology [33].

Based on plasma 25-hydroxyvitamin D levels before atorvastatin treatment, the participants, aged from 40 to 70 years, were assigned to one of three study groups. Group I included 26 women with vitamin D deficiency (plasma levels 25-hydroxyvitamin D between 25 and 50 nmol/L [between 10 and 20 ng/mL]), group II consisted of 26 women with vitamin D insufficiency (plasma levels 25-hydroxyvitamin D between 50 and 75 nmol/L [between 20 and 30 ng/mL]), while group III included women with normal vitamin D status (plasma levels 25-hydroxyvitamin D between 75 and 150 nmol/L [between 30 and 60 ng/mL]). For ethical reasons, women with 25-hydroxyvitamin D levels below 25 nmol/L (10 ng/mL) were not considered for enrollment, and group I included only women refusing additional vitamin D therapy.

The remaining exclusion criteria were as follows: 25-hydroxyvitamin D levels exceeding 150 nmol/L (60 ng/mL), diabetes, other endocrine disorders, grade 2 or 3 arterial hypertension, documented coronary artery disease, congestive heart failure, any acute vascular event in the past, autoimmune disorders, chronic inflammatory diseases, hematological disorders, impaired renal or hepatic function, malabsorption syndromes, other serious disorders, pregnancy or lactation. We also did not include women receiving drugs affecting plasma lipids, glucose homeostasis, other outcome measures, or interacting with statins and vitamin D.

**Figure 1:**
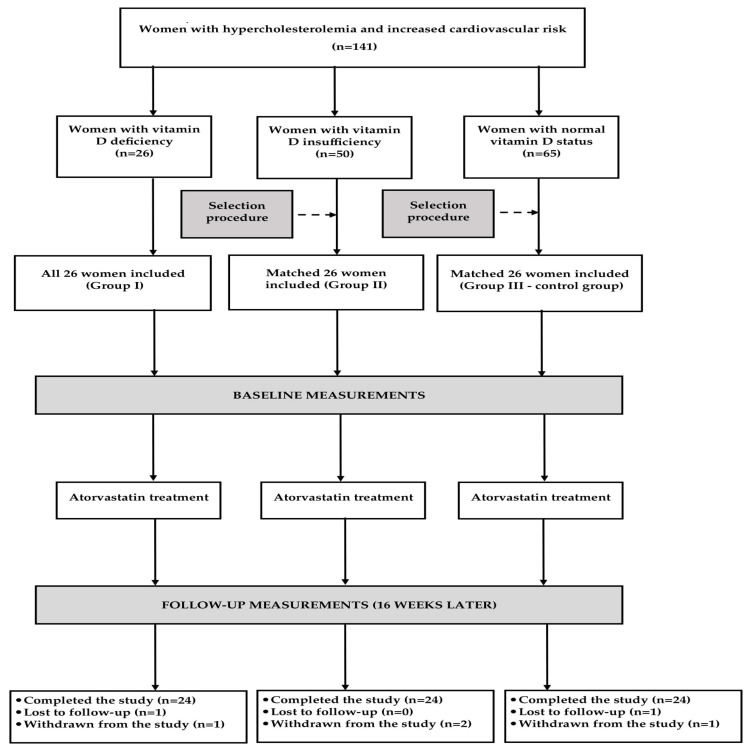
Flow the patients through the study.

All patients assigned to group I were enrolled. The remaining two groups were selected from larger cohorts of vitamin-insufficient and vitamin-sufficient women meeting the inclusion criteria (Figure 1). The intention of the selection procedure was to obtain three populations matched for age, body mass index (BMI), and plasma lipids. To minimize the influence of seasonal fluctuations in levels of 25-hydroxyvitamin D and the other outcome measures, similar proportions of women were recruited in spring (24%), summer (26%), autumn (23%), and winter (27%).

### 2.2. Study Design

All women participating in the study were treated with atorvastatin for 16 weeks. The starting daily dose of this drug was 20 mg. After 2 weeks, the initial dose was increased to 40 mg per day, and this final dose was administered until the end of the study. The medication was taken once daily at bedtime with a glass of water.

Vitamin D intake with food during the study was calculated based on analysis of dietary diaries completed by the participants. Total calciferol intake was calculated by adding intakes with food and supplemental tablets (if used). To minimize pharmacokinetic interactions between exogenous vitamin D and atorvastatin, vitamin D supplements were taken in the morning. During the study period, except for the scheduled increment in the atorvastatin dose, no changes in medication dosing were allowed. Patients’ adherence to the drug therapy was checked by interview and pill counting. Throughout the study, all groups of patients were also requested to follow the lifestyle modification requirements. Recommendations concerning physical activity were the same as in our previous cohort study [34]. Compliance with physical activity was verified by analysis of individual diaries in which the participants recorded all their activities. Non-opioid analgesic and antipyretic agents, non-steroidal anti-inflammatory drugs, antibiotics, antivirals, cough suppressants, antidiarrheal drugs, and laxatives or hypnotics were accepted if the duration of treatment period was not longer than 10 days, and the treatment was completed at least 30 days before the final visit. The withdrawal criteria included: consent withdrawal, serious adverse effects (meeting the Food and Drug Administration criteria for a serious adverse event [35]), other changes in pharmacotherapy and unsatisfactory medication adherence.

### 2.3. Laboratory Assays

Venous blood was taken from the antecubital vein via the venipuncture procedure on the first and last study day (before and after atorvastatin treatment). Blood was collected in the fasting state between 8.00 and 9.00 a.m. The assays were carried out in duplicate to verify the reproducibility of the results, and the obtained results were averaged. All measurements were performed by persons who were blinded to the study conditions. Fasting plasma levels of glucose and lipids, plasma creatine kinase (CK) activity, whole blood content of HbA_1c_, and urinary levels of creatinine and albumin were measured using a multi-analyzer COBAS Integra purchased from Roche Diagnostics (Basel, Switzerland). Plasma concentrations of 25-hydroxyvitamin D, insulin and homocysteine were assayed by direct chemiluminescence using acridinium ester technology (ADVIA Centaur XP Immunoassay System, Siemens Healthcare Diagnostics, Munich, Germany). Levels of high-sensitivity C-reactive protein (hs-CRP) were measured using an immunoassay with chemiluminescent detection (Immulite 2000XPi, Siemens Healthcare, Warsaw, Poland). Fibrinogen was assessed using the automated Clauss method, measuring the rate of fibrinogen to fibrin conversion in the presence of excess of thrombin. The homeostasis model assessment of insulin resistance (HOMA-IR) was calculated from fasting glucose (mmol/L) and insulin levels (mU/L) according to Matthews’ formula [36]. The albumin-to–creatinine ratio (UACR) was calculated by dividing total albumin concentration (μg) by creatinine concentration (mg) in urine.

### 2.4. Statistical Analysis

All continuous variables were log-transformed to achieve homogeneity of variance, and to minimize the impact of outliers. Comparisons between the different groups were performed using one-way analysis of variance followed by a Tukey’s post hoc test. Comparisons within the same group were made with a Student’s *t* test for paired data. A chi-square test was used to test categorical comparisons. The strength and direction of relationships between pairs of continuous variables were measured using Pearson’s r tests. Two-tailed *p*-values below 0.05 after correction for multiple comparisons were regarded as statistically significant.

## 3. Results

Four women meeting the withdrawal criteria and two women lost to follow-up prematurely terminated the study (Figure 1). The statistical analysis included the data of 72 patients (92%) who completed the study and were classified as adherent. There were no differences between the study groups in the intensity and duration of physical activity.

Before atorvastatin treatment, the study groups were similar in terms of age, smoking habits, BMI, and blood pressure. There were between-group differences in total daily calciferol intake and the percentage of patients on vitamin D supplementation (Table 1).

Before atorvastatin treatment, 25-hydroxyvitamin D concentrations were lowest in group I and highest in group III. HOMA-IR, HbA_1c_ and uric acid were higher in group I than in group III, while fibrinogen was higher in group I than in the remaining groups, but similar in groups II and III. HsCRP, homocysteine, and UACR were higher in group I than in groups II and III, and higher in group II than in group III. A 10-year risk of atherosclerotic events was higher in vitamin D-deficient than vitamin D-sufficient patients (Table 2).

In all study groups, atorvastatin decreased total cholesterol, LDL cholesterol, hsCRP, and a 10-year risk of atherosclerotic events. Only in group I did atorvastatin increase HOMA-IR, HbA_1c_, and CK. The decrease in homocysteine was observed in groups II and III, while the reduction in uric acid, fibrinogen, and UACR only in group III. At the end of the study, group I differed from the remaining groups in 25-hydroxyvitamin D, total cholesterol, LDL cholesterol, HOMA-IR, HbA_1c_, hsCRP, homocysteine, fibrinogen, UACR, 10-year risk of atherosclerotic events, and CK. After atorvastatin treatment, there were also differences between groups II and III in 25-hydroxyvitamin D, total and LDL cholesterol, uric acid, hsCRP, homocysteine, fibrinogen, UACR, and 10-year risk of atherosclerotic events, and between groups I and III in uric acid (Table 2). BMI and blood pressure did not change throughout the study. In groups II and III, the impact of atorvastatin on the outcome variables did not differ between users and non-users of calciferol supplements.

There were differences between groups III and the remaining groups in the percentage changes from the baseline in total cholesterol, LDL cholesterol, uric acid, hsCRP, homocysteine, fibrinogen, UACR, and the 10-year risk of atherosclerotic events; between group I and groups II and III in the percentage changes from the baseline in HOMA-IR, HbA_1c_, and CK, as well as between groups I and II in the percentage changes from the baseline in total cholesterol, LDL cholesterol, hsCRP, homocysteine, and the 10-year risk of atherosclerotic events (Table 2).

**Figure 2:**
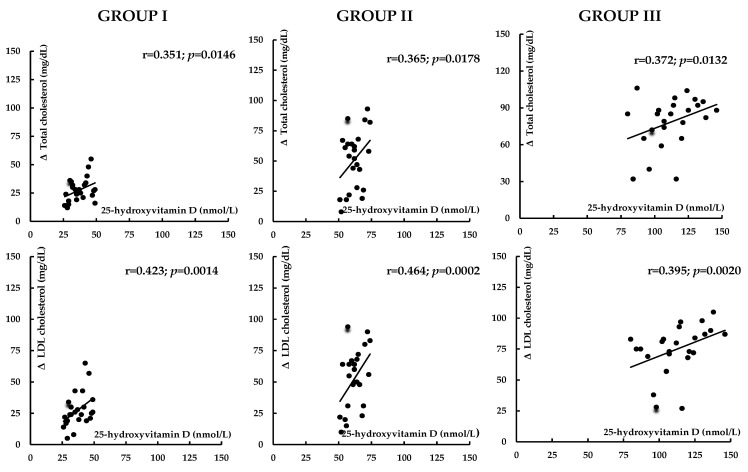
Correlations between the impact of atorvastatin on total and LDL cholesterol and vitamin D status. Group I: vitamin D-deficient women; Group II: vitamin D-insufficient women; Group III: vitamin D-sufficient women.

**Figure 3:**
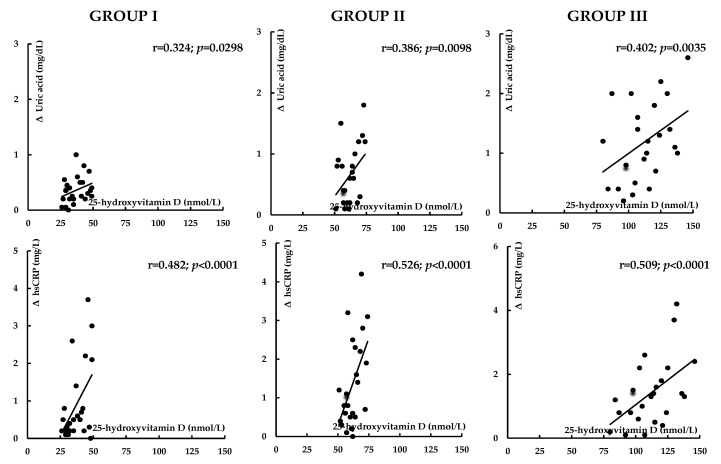
Correlations between the impact of atorvastatin on uric acid and hsCRP and vitamin D status. Group I: vitamin D-deficient women; Group II: vitamin D-insufficient women; Group III: vitamin D-sufficient women.

**Figure 4:**
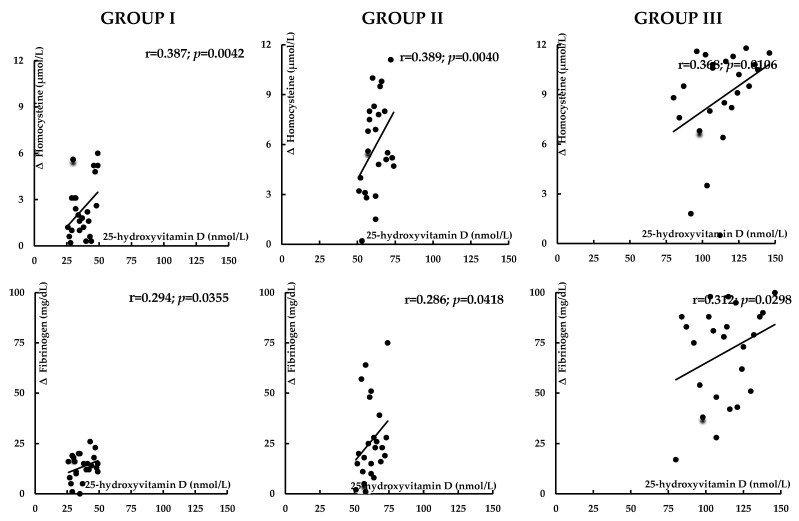
Correlations between the impact of atorvastatin on homocysteine and fibrinogen and vitamin D status. Group I: vitamin D-deficient women; Group II: vitamin D-insufficient women; Group III: vitamin D-sufficient women.

**Figure 5:**
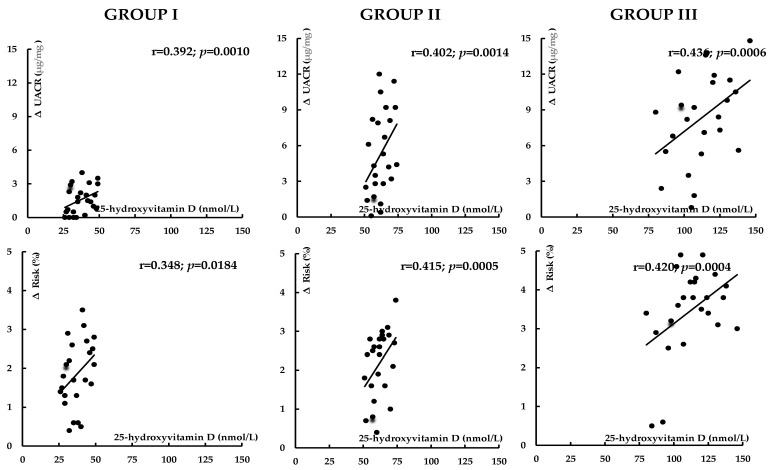
Correlations between the impact of atorvastatin on UACR and 10-year risk of atherosclerotic events. Group I: vitamin D-deficient women; Group II: vitamin D-insufficient women; Group III (control group): vitamin D-sufficient women.

Before atorvastatin treatment, 25-hydroxyvitamin D levels inversely correlated with hsCRP, homocysteine, and UACR. In group I, there were also inverse correlations between 25-hydroxyvitamin D levels and HOMA-IR, HbA_1c_, uric acid, and fibrinogen (Table 3). The impact of treatment on total and LDL cholesterol, uric acid, hsCRP, homocysteine, fibrinogen, UACR, and the 10-year risk of atherosclerotic events positively correlated with 25-hydroxyvitamin D concentrations (Figure 2, Figure 3, Figure 4 and Figure 5). In group I, but not in the remaining groups, there were positive correlations between the impact of atorvastatin on HOMA1-IR and HbA_1c_, and baseline hsCRP (r = 0.325, *p* = 0.0285; r = 0.298, *p* = 0.0415), and negative correlations between the changes in HOMA1-IR and HbA_1c_ and the changes in hsCRP (r = −0.342, *p* = 0.0214; r = −0.267, *p* = 0.0451). The impact of atorvastatin on the investigated variables did not correlate with daily vitamin D intake and the percentage of patients on vitamin D supplements. There were no correlations between changes in glucose homeostasis markers, uric acid, hsCRP, homocysteine, fibrinogen, and UACR and the effect on plasma lipids.

## 4. Discussion

Although the study groups were matched for plasma lipids, women with low vitamin D status were characterized by higher levels of cardiovascular risk factors than their peers with normal vitamin D homeostasis. The degree of this increase depended on the severity of the calciferol deficit, being more pronounced in case of vitamin D deficiency than insufficiency, and inversely correlating with 25-hydroxyvitamin D levels. These findings provide further evidence that low vitamin D status in women is likely to be associated with the earlier development and accelerated progression of atherosclerotic cardiovascular disease. In line with this hypothesis, vitamin D-deficient women were characterized by a higher 10-year risk of atherosclerotic events, despite no statistically significant differences in age, smoking, blood pressure, and cholesterol (total, HDL, and LDL), which are used in the calculation of this parameter [37]. The obtained results cannot be also explained by other aspects of baseline characteristics. Considering the association between hsCRP, homocysteine and UACR, and acute coronary and cerebral events [38,39,40], it seems that even 25-hydroxyvitamin D concentrations in the range between 50 and 75 nmol/L may predispose to unfavorable cardiovascular outcomes. Thus, even mild disturbances of calciferol homeostasis in middle-aged or elderly women may require effective supplementation, particularly if this risk is additionally increased by concomitant hypercholesterolemia. Considering higher values of HOMA-IR and HbA_1c_, individuals with 25-hydroxyvitamin D between 25 and 50 nmol/L may be also more prone to the development of type 2 diabetes, while our study does not provide convincing data that the risk of diabetes is increased in vitamin D-insufficient females.

The major finding of our study was that the strength of atorvastatin action on both plasma lipids and non-lipid risk factors depended on vitamin D homeostasis. These effects were most potent in vitamin D-sufficient women, in whom the drug reduced total and LDL cholesterol, as well as all investigated non-lipid risk factors (uric acid, hsCRP, homocysteine, fibrinogen, and UACR), irrespective of whether they were on vitamin D supplementation or received enough amounts of this micronutrient only with food. In the remaining groups of patients, these effects were much less pronounced and limited to a less pronounced decrease in total cholesterol, LDL cholesterol, and hsCRP, and in vitamin D-insufficient women additionally to a reduction in homocysteine. Thus, the vitamin D status of patients is an important determinant of both hypolipidemic and pleiotropic effects of atorvastatin in women. The obtained results are partially in agreement with our previous studies concerning atorvastatin action in males [32]. However, although vitamin D insufficiency attenuated non-lipid effects of atorvastatin in men, it did not affect its lipid-lowering properties. Thus, the interplay between sex hormones and vitamin D seems to modify the impact of atorvastatin on plasma lipids, but a high prevalence of low vitamin D status does not explain why lipid-lowering effects of HMG-CoA reductase inhibitors were found to be less pronounced in women than in men [41,42]. The obtained results are also in line with recent observations that vitamin D status may affect the distribution of body fat in low-dose statin users [43].

Between-group differences in atorvastatin action seem to reflect disturbances in calciferol homeostasis, which is supported by a range of observations. Firstly, the hypolipidemic and pleiotropic effects of atorvastatin inversely correlated with 25-hydroxyvitamin D concentration. Secondly, there were no intra-group differences in the strength of atorvastatin action between users and non-users of calciferol supplements. Thirdly, despite differences in total daily calciferol intake and in the percentage of patients supplemented with calciferol between the study groups, there were no correlations between these parameters and the impact of treatment on the outcome measures. Lastly, because of long intervals between taking vitamin D supplements and atorvastatin, the obtained results do not seem to be associated with pharmacokinetic interactions between exogenous calciferol and the HM-CoA reductase inhibitor.

The effect of statins on vitamin D status is inconclusive, as statins were found to increase [44,45] or have a neutral effect [46,47] on 25-hydroxyvitamin D. What is more, in the meta-analysis by Mazidi et al. [48] the impact depended on the study design. HMG-CoA reductase inhibitors increased 25-hydroxyvitamin D in randomized controlled trials, while the opposite effect (decrease) was observed in non-randomized studies [43]. From a theoretical point of view, HMG-CoA reductase inhibitors should worsen vitamin D homeostasis because they reduce the production of the cholecalciferol precursor (7-dehydrocholesterol) and because LDL cholesterol plays a role as a calciferol carrier [49]. In all study groups, however, 25-hydroxyvitamin D remained at stable levels, which was in line with our observations concerning males [32]. This may suggest that the impact on synthesis and transport is counterbalanced by other mechanisms (particularly by the inhibitory effect of atorvastatin treatment on the hepatic metabolism of calcitriol [50]).

Between-group differences in the impact of statin therapy on the lipid profile are in line with the results obtained by Pérez-Castrillón et al. [51], who did not observe changes in plasma lipids in myocardial infarction survivors receiving atorvastatin treatment if 25-hydroxyvitamin D levels were below 30 nmol/L. These differences are also in agreement with the findings by Catalano et al. [52], who found that calcifediol potentiated the impact of atorvastatin on LDL and HDL cholesterol in postmenopausal women. More pronounced hypolipidemic properties of atorvastatin in women with undisturbed calciferol homeostasis and additive effects of atorvastatin and vitamin D may be explained by a strong inhibitory concentration-dependent effect of calciferol and its metabolites on the activity of HMG-CoA, the main mechanism of statin action [53].

It is also worth underlining that the calciferol status determined statin action on glucose homeostasis. Unlike vitamin D-sufficient and vitamin D-insufficient women, in whom this effect was neutral, the atorvastatin treatment of vitamin D-deficient women was accompanied by worsening insulin sensitivity and by higher levels of HbA_1c_, which is a marker of average glucose concentrations over the past 2–3 months [54]. Thus, it cannot be excluded that an increased risk of type 2 diabetes is at least partially related to low vitamin D status, and may not be observed after normalization of calciferol homeostasis. Interestingly, the changes in both HOMA-IR and HbA_1c_ in vitamin D-deficient women positively correlated with baseline hsCRP levels and negatively correlated with treatment-induced changes in hsCRP, which, in turn, were related to 25-hydroxyvitamin D levels. However, there were no direct correlations between glucose homeostasis markers and 25-hydroxyvitamin D concentration. These findings indirectly suggest that the unfavorable impact of atorvastatin on glucose homeostasis in vitamin D-insufficient individuals may be determined by low-grade inflammation.

Only one participant was dropped from the study because of serious adverse muscular effects. This finding may be related to age (young or middle-aged participants), ethnic considerations (Polish Caucasians), the lack of patients with concomitant disorders (particularly hypothyroidism and disorders associated with low body mass), and rare (usually short-term) use of other drugs, resulting from the inclusion and exclusion criteria. Some studies emphasize the link between statin-associated myopathy and low-vitamin D status [55,56], although other data speak against such a relationship [57]. In the current study, atorvastatin increased CK activity only in vitamin D-deficient women, but posttreatment values were almost always within the reference range. Moreover, between-group differences in this parameter were observed after (but not before) atorvastatin treatment. These findings, partially explaining the inconsistent results of previous studies [55,56,57], suggest that the risk of atorvastatin-induced adverse muscular effects in women with low vitamin D status but without other risk factors for myopathy is low and usually limited to an asymptomatic increase in CK.

The results of our study also allow us to draw other practical conclusions. Firstly, vitamin D supplementation in middle-aged and elderly women should be directed not only at normalizing calcium homeostasis and bone structure but also at improving cardiometabolic health. Secondly, it seems justified to assess 25-hydroxyvitamin D levels in all patients who are candidates for therapy with atorvastatin and likely also with other HMG-CoA reductase inhibitors. Thirdly, it seems reasonable to periodically assess vitamin D status during treatment with these agents. Fourthly, even asymptomatic individuals with low vitamin D status receiving statin therapy may require vitamin D supplementation, and the target 25-hydroxyvitamin D levels should be at least 75 nmol/L. Lastly, vitamin D-sufficient middle-aged or elderly women do not seem to be at higher risk of development of cardiometabolic disorders if they do not have other risk factors.

There are numerous study limitations that should be acknowledged. The small number of participants limits the reliability and utility of our findings, making it difficult to draw definite conclusions. Owing to the broad age range, there can be some differences between premenopausal and postmenopausal women. It is uncertain whether the benefits seen in our study represent a class effect of statin therapy or are specific to atorvastatin. Measurement of only surrogate outcomes provides only probabilistic answers, but does not guarantee clinically relevant findings. The results might have been impacted by systematic errors (selection biases, information biases, and confounding). The study design does not allow us to demonstrate the beneficial effects of vitamin D supplementation. Owing to no between-group differences, the intensity and duration of physical activity do not seem to explain the obtained results. However, the type of physical activity might have had some indirect impact on our findings by causing differences in sunlight exposure. It remains to be elucidated whether low vitamin D status impairs the lipid-lowering and pleiotropic effects of high-dose statin therapy. Lastly, because of the exclusion criteria, the interaction between vitamin D deficiency and insufficiency and atorvastatin action in individuals with documented cardiovascular disease or diabetes requires further investigation.

## 5. Conclusions

Despite matching the study groups for age, BMI, and plasma lipids, women with low vitamin D status, particularly those with vitamin D deficiency, were characterized by increased levels of cardiovascular risk factors and a higher 10-year risk of atherosclerotic events. The hypolipemic effects of atorvastatin, though observed irrespective of the calciferol status of the patients, were most pronounced in women with normal vitamin D homeostasis. Low vitamin D status impaired the pleiotropic effects of atorvastatin proportionally to its degree. Moreover, women with 25-hydroxyvitamin D between 25 and 50 nmol/L were characterized by deterioration of glucose homeostasis in response to atorvastatin treatment. The obtained results suggest that low vitamin D status has a multifaceted negative impact on atorvastatin action in middle-aged or elderly women and may argue that the measurement of 25-hydroxyvitamin D and eventual vitamin D supplementation should be recommended to all women who are candidates for statin therapy. Given the limited sample size and other methodological limitations, larger randomized controlled trials are needed to verify our findings. Future studies should also help identify the molecular mechanisms of interactions between HMG-CoA reductase inhibitors and vitamin D homeostasis.

## Figures and Tables

**Table 1:** Baseline characteristics of both study groups.

Variable	Group I	Group II	Group III
**Number** (n)	24	24	24
**Age** (years)	60 ± 8	59 ± 8	58 ± 9
**Smokers** (%)/**Number of cigarettes a day** (n)**/Duration of smoking** (years)	58/10 ± 6/29 ± 8	54/9 ± 5/27 ± 8	50/11 ± 7/30 ± 9
**BMI** (kg/m^2^)	27.4 ± 5.0	26.8 ± 5.2	26.4 ± 4.9
**Systolic blood pressure** (mmHg)	132 ± 16	128 ± 17	126 ± 19
**Diastolic blood pressure** (mmHg)	86 ± 7	85 ± 7	84 ± 7
**Total daily calciferol intake** (µg)	8 ± 3 ^&$^	13 ± 4 ^$^	29 ± 8
**Users of calciferol supplements** (%)	4 ^&$^	29 ^$^	50

Group I: vitamin D-deficient women; Group II: vitamin D-insufficient women; Group III (control group): vitamin D-sufficient women. Except for the percentages of smokers and users of calciferol supplements, the data are shown as the mean ± standard deviation. ^&^ *p* < 0.05 vs. group II, ^$^ *p* < 0.05 vs. group III.

**Table 2:** The effect of atorvastatin on the investigated variables in the study population.

Variable	Group I	Group II	Group III
**25-hydroxyvitamin D** (nmol/L)			
*Before atorvastatin treatment*	37.2 ± 6.5 ^&$^	62.0 ± 6.7 ^$^	112.1 ± 18.7
*After atorvastatin treatment*	38.5 ± 6.4 ^&$^	63.8 ± 6.2 ^$^	118.1 ± 14.6
*Percentage change from baseline*	3 ± 8	2 ± 8	5 ± 10
**Total cholesterol** (mg/dL)			
*Before atorvastatin treatment*	232 ± 41	236 ± 46	241 ± 50
*After atorvastatin treatment*	206 ± 38 *^&$^	184 ± 35 *^$^	164 ± 29 *
*Percentage change from baseline*	−11 ± 14	−22 ± 12 ^#^	−32 ± 16 ^#&^
**HDL cholesterol** (mg/dL)			
*Before atorvastatin treatment*	48 ± 10	50 ± 10	51 ± 9
*After atorvastatin treatment*	49 ± 12	50 ± 11	53 ± 11
*Percentage change from baseline*	2 ± 5	0 ± 8	4 ± 10
**LDL cholesterol** (mg/dL)			
*Before atorvastatin treatment*	146 ± 23	150 ± 20	153 ± 25
*After atorvastatin treatment*	120 ± 28 *^&$^	98 ± 23 *^$^	78 ± 19 *
*Percentage change from baseline*	−18 ± 10	−35 ± 17 ^#^	−49 ± 20 ^#&^
**Triglycerides** (mg/dL)			
*Before atorvastatin treatment*	176 ± 69	171 ± 78	168 ± 74
*After atorvastatin treatment*	168 ± 74	161 ± 73	153 ± 64
*Percentage change from baseline*	−3 ± 14	−6 ± 15	−9 ± 18
**Glucose** (mg/dL)			
*Before atorvastatin treatment*	93 ± 12	92 ± 13	90 ± 11
*After atorvastatin treatment*	95 ± 14	93 ± 14	90 ± 13
*Percentage change from baseline*	2 ± 6	1 ± 5	0 ± 5
**HOMA-IR**			
*Before atorvastatin treatment*	2.6 ± 0.9 ^$^	2.3 ± 0.9	1.9 ± 0.8
*After atorvastatin treatment*	3.2 ± 1.0 *^&$^	2.5 ± 1.0	2.0 ± 0.8
*Percentage change from baseline*	23 ± 20 ^&$^	9 ± 15	5 ± 10
**HbA_1c_** (%)			
*Before atorvastatin treatment*	5.3 ± 0.5 ^$^	5.1 ± 0.5	5.0 ± 0.4
*After atorvastatin treatment*	5.6 ± 0.4 *^&$^	5.2 ± 0.5	5.0 ± 0.4
*Percentage change from baseline*	6 ± 7 ^&$^	2 ± 5	0 ± 5
**Uric acid** (mg/dL)			
*Before atorvastatin treatment*	5.0 ± 1.7 ^$^	4.6 ± 1.8	4.1 ± 1.3
*After atorvastatin treatment*	4.7 ± 1.6 ^$^	4.0 ± 1.4 ^$^	3.0 ± 1.1 *
*Percentage change from baseline*	−6 ± 16	−15 ± 20	−27 ± 19 ^#&^
**hsCRP** (mg/L)			
*Before atorvastatin treatment*	3.8 ± 1.4 ^&$^	3.0 ± 1.1 ^$^	2.3 ± 0.8
*After atorvastatin treatment*	3.0 ± 0.8 *^&$^	1.7 ± 1.0 *^$^	1.0 ± 0.6 *
*Percentage change from baseline*	−21 ± 23	−43 ± 25 ^#^	−57 ± 20 ^#&^
**Homocysteine** (μmol/L)			
*Before atorvastatin treatment*	34.1 ± 11.9 ^&$^	27.5 ± 9.8 ^$^	20.4 ± 8.8
*After atorvastatin treatment*	31.8 ± 10.2 ^&$^	21.6 ± 9.4 *^$^	11.7 ± 8.4 *
*Percentage change from baseline*	−7 ± 14	−21 ± 22 ^#^	−43 ± 26 ^#&^
**Fibrinogen** (mg/dL)			
*Before atorvastatin treatment*	443 ± 93 ^&$^	380 ± 102	346 ± 88
*After atorvastatin treatment*	429 ± 87 ^&$^	358 ± 90 ^$^	275 ± 76 *
*Percentage change from baseline*	−3 ± 10	−6 ± 12	−21 ± 18 ^#&^
**UACR** (μg/mg)			
*Before atorvastatin treatment*	35.0 ±11.3 ^&$^	27.1 ± 11.0 ^$^	20.4 ± 8.2
*After atorvastatin treatment*	34.0 ± 12.4 ^&$^	22.1 ± 8.7 ^$^	11.8 ± 5.8 *
*Percentage change from baseline*	−3 ± 20	−18 ± 35	−42 ± 40 ^#&^
**10-year risk of atherosclerotic events** (%)			
*Before atorvastatin treatment*	10.3 ± 2.6 ^$^	9.2 ± 2.8	8.9 ± 2.0
*After atorvastatin treatment*	8.4 ± 2.5 *^&$^	7.0 ± 2.2 *^$^	5.3 ± 2.0 *
*Percentage change from baseline*	−18 ± 9	−24 ± 11 ^#^	−40 ± 14 ^#&^
**CK** (U/L)			
*Before atorvastatin treatment*	70 ± 40	62 ± 30	58 ± 28
*After atorvastatin treatment*	110 ± 48 *^&$^	80 ± 42	68 ± 35
*Percentage change from baseline*	57 ± 46 ^&$^	29 ± 42	17 ± 40

Group I: vitamin D-deficient women; Group II: vitamin D-insufficient women; Group III (control group): vitamin D-sufficient women. The data are shown as the mean ± standard deviation. * *p* < 0.05 vs. before atorvastatin treatment in the same group, ^#^ *p* < 0.05 vs. group I, ^&^ *p* < 0.05 vs. group II, ^$^ *p* < 0.05 vs. group III.

**Table 3:** Baseline correlations between the outcome measures.

Correlated Variables		Group I	Group II	Group III
HOMA-IR	25-hydroxyvitamin D	−0.386 *	−0.223	−0.202
HbA_1c_	25-hydroxyvitamin D	−0.345 *	−0.207	−0.195
Uric acid	25-hydroxyvitamin D	−0.285 *	−0.205	−0.186
hsCRP	25-hydroxyvitamin D	−0.523 *	−0.490 *	−0.465 *
Homocysteine	25-hydroxyvitamin D	−0.486 *	−0.423 *	−0.402 *
Fibrinogen	25-hydroxyvitamin D	−0.351 *	−0.258	−0.224
UACR	25-hydroxyvitamin D	−0.398 *	−0.370 *	−0.311 *

Group I: vitamin D-deficient women; Group II: vitamin D-insufficient women; Group III (control group): vitamin D-sufficient women. The data represent the correlation coefficients (r-values). Statistical significance is marked by asterisks.

## Data Availability

The data that support the findings of this study are available from the corresponding author upon reasonable request.
